# Formulation of Gels and Emulgels with *Malus domestica* Borkh: Apple Extracts and Their Biopharmaceutical Evaluation In Vitro

**DOI:** 10.3390/antiox11020373

**Published:** 2022-02-12

**Authors:** Aurita Butkeviciute, Kristina Ramanauskiene, Valdimaras Janulis

**Affiliations:** 1 Department of Pharmacognosy, Lithuanian University of Health Sciences, Sukileliu Ave. 13, LT-50162 Kaunas, Lithuania; Valdimaras.Janulis@lsmuni.lt; 2 Department of Clinical Pharmacy, Lithuanian University of Health Sciences, Sukileliu Ave. 13, LT-50162 Kaunas, Lithuania; Kristina.Ramanauskiene@lsmuni.lt

**Keywords:** antioxidants, apple, polyphenols, topical preparations

## Abstract

Phenolic compounds that estimate apple extracts with multifaceted biological effects are potentially valuable for protection against skin disorders. The purpose of our research was to formulate gels and emulgels containing a complex of phenolic compounds of apple extracts and to perform a biopharmaceutical evaluation of semi-solid pharmaceutical forms, determining their antioxidant activity in vitro. HPLC analyses of phenolic compounds were performed. The total amount of phenolic compounds found in the sample of apples from the ‘Paprastasis antaninis’ cultivar was 1455.5 ± 72.8 µg/g. The release of phenolics from gels and emulgels was assessed by Franz-type diffusion cells. The in vitro release test revealed that phenolic compounds were released from the gel (G1–G6) formulations (70.6–73.8%) compared to the amounts (77.2–83.9%) released from the emulgel (E1–E6) formulations. The largest amount (83.9%) of phenolic compounds was released from the E5 formulation, while the smallest amounts (70.6%) were released from the formulations G3 and G5. The antioxidant activity evaluated by the DPPH and FRAP methods observed in all gel (G1–G6) and emulgel (E1–E6) formulations after 6 h were the strongest, compared to the activities observed in the formulations after 2 or 4 h. Gels and emulgels, which are rich in apple extracts, have strong antioxidant properties and may be promising choices for the development of new, innovative pharmaceutical forms or cosmetics.

## 1. Introduction

*Malus domestica* Borkh. apples are among the most cultivated fruits in the world, with high nutritional value [1,2]. In the human diet, apples are an important component, and their nutritional value is estimated according to phenolic acids, flavonoids, triterpenes, organic acids, vitamins, micro- and macro-elements, and dietary fiber [3,4]. Phenolics are a group of bioactive compounds that determine the antioxidant activity of apple extracts by acting as reducing agents and binding free radicals, thus protecting macromolecular structures from the harmful effects of free radicals [2,4,5]. Natural phenolics with anti-inflammatory, antioxidant, antibacterial, immune-system-promoting, and photoprotective effects are potentially valuable in wound healing therapies, in the prevention of chronic inflammatory skin diseases and skin disorders caused by viral, bacterial, or fungal infections, premature aging, pigmentation disorders, and in the protection of skin from UV radiation [5,6,7]. Currently, there is great consumer interest in natural products, which include compounds obtained from fruits, plants, and herbs [5]. Consequently, due to their multifaceted biological effects, the phenolic compounds found in apples are interesting candidates for the development of topical dosage forms for pharmaceutical and medical applications.

As fruit components, phenolic compounds are not fully released and the released compounds are poorly absorbed. The bioavailability of phenolic compounds is not particularly high; compounds with a lower molecular weight are more readily absorbed in the gastrointestinal tract [8]. Previous studies have revealed that various groups of phenolics are absorbed at a rate of 0.3–43.0% and that the metabolite contents circulating in the plasma can be low [9]. Chlorogenic acid absorption is about 33.0%; the highest levels of chlorogenic acid are absorbed in the large intestine, while (+)-catechin and (−)-epicatechin are both absorbed by small intestinal epithelial cells [10]. The bioavailability of flavonoids depends on their physicochemical properties, including the degree of polymerization, glycosylation or molecular properties, their polarity and interaction with nutrients and the proteins in their cells [11]. For flavonoids with complex structures and larger molecular weights, bioavailability may be even lower [12,13]. Biotransformation affects phenolics’ physical properties; therefore, they are more soluble in water and have better antioxidant activities. Some metabolites of phenolics have the same antioxidant properties as their precursors. However, in general, metabolites are weaker antioxidants because of the modifications of their catechol and phenol groups. Data about the concentration of flavonoids in human tissues are not available, but it is unlikely that flavonoids have a significant impact on the antioxidant activity in cells [14]. There are data suggesting that the route of administration, the release of a dosage form, and absorption are known to affect bioavailability [15].

The administration of topical preparations depends on the type and severity of disease. For skin disorders, the topical route is preferred. The topical preparation delivery system has several advantages, such as the ability to introduce the active substance to a required location and its avoidance of the gastrointestinal tract [16]. In addition, local deliveries ensure increased bioavailability, the bypassing of first-pass metabolism and consistent delivery for an extended time [17,18]. One of the important steps in formulating a semi-solid form is the choice of the pharmaceutical form category. In our research, semi-solid pharmaceutical forms, gel and emulgel, which are emerging techniques in topical preparation delivery systems, were selected [19]. Emulgels are simply emulsions that are gelled by mixing with a gelling material; thus, emulsions can be of either the oil-in-water or the water-in-oil type, subject to the purpose of use. In an emulsion, the entrapped active substance is slowly released from the internal phase through the external phase and is slowly absorbed through the skin [20]. Further, gels and emulgels have excellent properties, such as being thixotropic and readily detachable, spreading readily, being non-oily, negotiating with numerous excipients, and being non-staining, miscible, transparent, and biofriendly. Emulgels have better cutaneous penetration and stability than other available topical preparations [21]. Topical preparations, such as creams or ointments, have many limitations, such as a lower spreading coefficient, less penetration through the stratum corneum and less patient compliance due to stickiness or the need to apply with rubbing [21]. The scientific research provides data suggesting that creams have the challenge of phase inversion and breaking, while ointments may have rancidity [20]. Similarly, gels have the limitation of delivering hydrophobic active substances [21]. When one of the semi-solid forms components is weakly water-soluble, emulgels may be a better choice [22].

Topical formulations with fruit extracts and biologically active compounds have become popular due to their natural origin and non-toxicity, and they are used in the treatment of a variety of skin disorders. Even though there are many ointments and creams on the market that use various plant extracts, apple extracts with a complex of phenolic compounds are still rarely used in the production of emulgels and gels. During our research, we selected excipients and modeled gels and emulgels with a complex of phenolic compounds found in apple extracts and performed a biopharmaceutical evaluation of these semi-solid pharmaceutical forms in vitro. We conducted an in vitro evaluation of the antioxidant activity of gels and emulgels containing a complex of phenolic compounds of apple extracts. The results of this research will enable the development and production of new, innovative, semi-solid pharmaceutical forms with a complex of phenolic compounds of apple extracts.

The aim of our study was to formulate gels and emulgels containing a complex of phenolic compounds of apple extracts and to perform a biopharmaceutical evaluation of semi-solid pharmaceutical forms, determining their antioxidant activity in vitro.

## 2. Materials and Methods

### 2.1. Plant Materials

The apple cultivar ‘Paprastasis antaninis’ was used in this study. The apple trees were grown in the experimental orchard of the Institute of Horticulture, Lithuanian Research Centre for Agriculture and Forestry, Babtai, Lithuania (55°60′ N, 23°48′ E). The altitude of Babtai town is 57 m above sea level. The apples, harvested in September 2020, were immediately lyophilized and used for the study.

### 2.2. Chemicals and Solvents

All solvents, reagents, and standards used were of analytical grade. The standards used in the HPLC analysis were the following: hyperoside, rutin, quercitrin, phloridzin, procyanidin B1, procyanidin B2 and chlorogenic acid, obtained from Extrasynthese (Genay, France); reynoutrin, (+)-catechin and (–)-epicatechin, purchased from Sigma-Aldrich GmbH (Buchs, Switzerland); and avicularin and isoquercitrin, obtained from Chromadex (Santa Ana, CA, USA). The chemicals applied in the modeling of semi-solid pharmaceutical forms were glycerin, castor oil, sorbitan monolaurate (Span 20), polyethylene glycol sorbitan monolaurate (Tween 20) and sodium hydroxide (NaOH), obtained from Sigma-Aldrich Chemie GmbH (Steinheim, Germany). Carbomer 980 was obtained from Lubrizol (Wickliffe, OH, USA). The reagents used in the antioxidant activity assay were 6-hydroxy-2,5,7,8-tetramethylchroman-2-carboxylic acid (Trolox), 2,2-diphenyl-1-picrylhydrazyl (DPPH) and sodium acetate, acquired from Scharlau (Barcelona, Spain). Iron (III) chloride hexahydrate (FeCl_3_ × 6H_2_O) was purchased from Vaseline-Fabrik Rhenania (Bonn, Germany) and 2,4,6-tri(2-pyridyl)-s-triazine (TPTZ) was obtained from Carl Roth (Karlsruhe, Germany).

### 2.3. Preparation of the Apple Lyophilizate

The apples were cut into slices of equal size (up to 1 cm in thickness), and the stalks and the seeds were removed. The apple slices with peel were immediately frozen in a freezer (at −35 °C) with air circulation. The apple samples were lyophilized with a ZIRBUS sublimator 3 × 4 × 5/20 (ZIRBUS technology, Bad Grund, Germany) at a pressure of 0.01 mbar (condenser temperature, −85 °C). The lyophilized apple slices were ground to fine powder (particle size about 100 µm) by using a knife mill Grindomix GM 200 (Retsch, Haan, Germany) [23].

### 2.4. Production of Dry Apple Extracts

The dry extracts of phenolic compounds were prepared by applying the method described by Butkeviciute et al. [23].

### 2.5. HPLC-PDA Analysis for the Characterization of Phenols

Phenolic profiles in the apple extracts were determined by using the HPLC method developed by Liaudanskas et al. [24].

### 2.6. Formulation of Semi-Solid Pharmaceutical Forms and Their Biopharmaceutical Evaluation

#### 2.6.1. Preparation of Gels, Emulsions and Emulgels

Gels were formulated using different concentrations of carbomer. The appropriate amount of carbomer was weighed and mixed with the proper content of purified water; the mixtures were kept at room temperature for 24 h. Next, 10% (v/v) sodium hydroxide was added dropwise to the formulations until the pH value reached 6.2 and homogeneous gel forms were obtained. Glycerin, castor oil, Span 20, Tween 20, and an appropriate amount of water were mixed with a magnetic stirrer IKA^®^ C-MAG HS 7 (IKA^®^-Werke GmbH & Co. KG, Staufen Im Breisgau, Germany) until homogeneous emulsions were prepared. Equal contents of gels (10 g) and emulsions (10 g) were mixed with a magnetic stirrer until emulgels of homogeneous structure were formed. We chose to add 1% aqueous dry extracts of phenolic compounds to each prepared gel and emulgel formulation. All the experimental formulations were stored in a refrigerator (at 5 °C) (Table 1).

#### 2.6.2. Evaluation of Physiochemical Properties of Gels, Emulsions and Emulgels

The pH values of the gels and emugels were determined at room temperature with a pH meter (766 with a Knick SE 104N electrode, Berlin, Germany). The pH meter was calibrated with buffer solutions at pH 4.0–7.0. The viscosity of experimental formulations was determined with a vibrating viscometer (Vibro viscometer SV-10, A&D Company ltd, Tokyo, Japan) [25]. Approximately 40 g of the tested semi-solid preparation was placed in the viscometer cell; the sensor plate was then lowered and the dynamic viscosity was measured. Measurements were performed at room temperature.

#### 2.6.3. In Vitro Studies of Release from Gel and Emulgel Formulations

The evaluation of the release of phenolic compounds from gel and emulgel formulations was performed using Franz-type diffusion cells with natural cellulose dialysis membranes. The receptor compartment contained an ethanol–water mixture at a ratio of 1:1, the temperature being 37.0 ± 0.5 °C. The donor compartment contained 1 g of gels and emulgels. A dialysis tubing cellulose membrane (Sigma Aldrich, St. Louis, MI, USA) was placed between the donor and the receptor compartments. The receptor medium was stirred using the hotplate magnetic stirrer IKAMAG C-MAG HS7 (IKA-Werke GmbH & Co. KG, Staufen, Germany) [25]. The 1 mL samples of the acceptor medium wafter were taken after 1 h, 2 h, 3 h, 4 h and 5 h and the last samples were obtained after 6 h. The samples were analyzed by applying the HPLC-PDA method.

### 2.7. Evaluation of Antioxidant Activity

The antioxidant activity of the dry apple extract was assessed by two different in vitro spectrophotometric assays using a spectrophotometer (Spectronic CamSpec M550, Garforth, UK). The DPPH free radical scavenging assay was assessed when 3000 µL of the DPPH reagent was mixed with 10 µL of the apple extract. A decrease in absorbance was measured after 30 min at 517 nm wavelength [26]. For the determination of the reducing activity by the FRAP method (Ferric Ion Reducing Antioxidant Power), the FRAP solution included TPTZ (0.01 M dissolved in 0.04 M HCl), FeCl_3_ × 6H_2_O (0.02 M in water) and an acetate buffer (0.3 M, pH 3.6) (ratio 1:1:10). In the course of research, 3000 µL of FRAP solution was mixed with 10 µL of the apple extract. An increase in absorbance was estimated at 593 nm wavelength [27]. The estimation of the antioxidant activity of the apple extract was calculated using a Trolox calibration curve, which calculates a µM Trolox equivalent (TE) per gram of the absolute dry weight (DW). The TE was appreciated according to the formula TE = c × V/m (µM/g), where c is the concentration of Trolox detected from the calibration curve (in µM), V is the volume of the apple extract (in L) and m is the weight (precise) of the lyophilized apple powder (in g).

### 2.8. Statistical Analysis

The statistical analysis of the research data was performed by using Microsoft Office Excel 2013 (Microsoft, Redmond, WA, USA) and SPSS 25.0 (SPSS Inc., Chicago, IL, USA) computer software. All the results were presented as means of the results of three consecutive tests and their standard deviations. To estimate the variance in the quantitative composition, we calculated the coefficient of variation. ANOVA was used to determine the differences between the compared data that were statistically significant. If the variances of independent variables were estimated to be equal, Tukey’s multiple comparison test was applied. The differences were evaluated as statistically significant at *p* < 0.05. The comparison of the released content of phenolic compounds between the experimental formulations was performed by applying the hierarchical cluster analysis, using the squared Euclidean distance.

## 3. Results and Discussion

### 3.1. Phenolic Content of Apple Extracts

**Figure 1:**
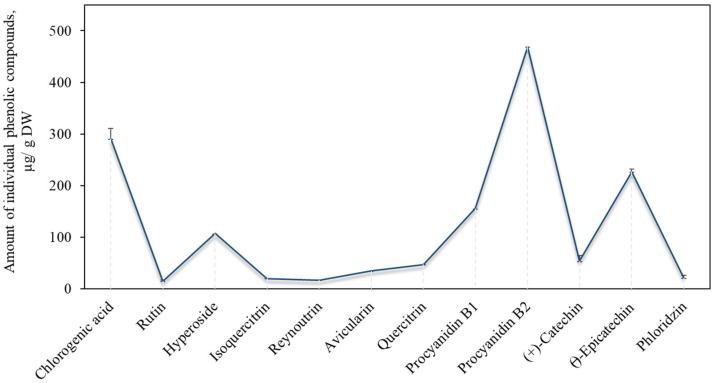
Content of chlorogenic acid, flavonols, flavan-3-ols and phloridzin.

During the first stage of the study, we found variations in the qualitative and quantitative composition of the phenolic compounds in the apple extracts. The phenolics were identified and quantified in the apple samples: flavonols (rutin, hyperoside, isoquercitrin, reynoutrin, avicularin and quercitrin), flavan-3-ols (procyanidin B1, procyanidin B2, (+)-catechin and (−)-epicatechin,), a dihydrochalcone (phloridzin) and a phenolic acid (chlorogenic acid). The total amount of phenolic compounds found in the sample of the apples from the ‘Paprastasis antaninis’ cultivar was 1455.5 ± 72.8 µg/g. Compounds of the flavan-3-ol group predominated among all the identified phenolic compounds. The total amount of the compounds of this group (904.8 ± 45.2 µg/g) accounted for 62.2% of all the phenolic compounds identified. The variation in the content of the individual flavan-3-ol compounds in the apple sample is shown in Figure 1. Oligomeric (procyanidin B1 and procyanidin B2) flavan-3-ol compounds predominated in the apple samples. Belviso et al. established that the amount of procyanidin B2 in apples samples ranges from 18.0 µg/g to 2090.0 µg/g [28]. The results of the study confirm that procyanidins are among the most widely determined compounds of the flavan-3-ol group in apples [28].

The total amount of flavonols identified and quantified in the apple extracts was 238.8 ± 11.9 µg/g, which accounted for 16.4% of the total phenolic compounds detected (Figure 1). The flavonols identified and quantified in the apple extracts can be ranked by their amount in the following ascending order: rutin < reynoutrin < isoquercitrin < avicularin < quercitrin < hyperoside. Hyperoside was the prevailing compound among flavonols in the apples from the cultivar selected for this study. Our results are consistent with those of previously published studies [29]. The content of the identified and quantified chlorogenic acid was 290.4 ± 14.5 µg/g, which accounted for 20.0% of the total phenolic compounds detected (Figure 1). Tsao et al. determined that chlorogenic acid reaches 21.0–90.0% of the total content of phenolics in apples. [30]. Similarly, Rana et al. determined that the level of chlorogenic acid in apples ranges from 106.8 µg/g to 198.9 µg/g [31]. The dihydrochalcone group compound phloridzin was also identified in the apple extracts. Its content in the samples was 21.5 ± 1.1 µg/g, accounting for only 1.5% of the total phenolic compounds found in the apple samples (Figure 1). Piccolo et al. established that the amount of phloridzin in apples varies from 10.0 µg/g to 50.0 µg/g, confirming the results of our study [32]. The dihydrochalcone group compounds can be chosen as chemotaxonomic markers in the taxonomy of apple species, as well as for the appreciation of apple products and their quality [33].

Phenolics reveal a series of biological activities that affect human health. Because of their natural origin and weak toxicity, phenolics could reduce the causes and effects of skin aging, wrinkles, acne and other skin disorders [34].

### 3.2. Formulation and Physicochemical Characteristics of Semi-Dolid Forms

The attempt to cure skin diseases has been the driving force in the discovery of various medicines and delivery systems. The selection of the appropriate delivery system depends on many factors, such as the hydrophobicity or hydrophilicity of the bioactive substances, the route of delivery into the body, and the desired release profile of the compound [35]. One of the important steps in order to obtain a therapeutic response to bioactive compounds required for treatment is the selection of the appropriate pharmaceutical form. Consequently, following an analysis of the qualitative and quantitative composition of the active compounds in apple extracts, in the next stage of the study, we produced six gels and emulgels of different concentrations containing 1% of the aqueous apple extract (AE), the organoleptic characteristics and physicochemical parameters of which are presented in Table 2.

Semi-solid pharmaceutical forms must have adequate performance: they should preserve skin barrier homeostasis and the integrity of the stratum corneum; they should provide antimicrobial protection; and they should cause no skin irritation [36]. The pH of the modeled formulations should be similar to that of the skin, i.e., from 4.1 to 7.0 [37,38]. When the pH of the preparation is between 9.0 and 10.0, the skin is irritated, the properties of the protective barrier are lost, and suitable conditions are created for bacteria to multiply [39,40]. Scientific research suggests that preparations with a neutral or acidic pH (pH ≤ 7.0) are safer than preparations that are alkaline. Semi-solid preparations with an alkaline pH may cause swelling of the skin, may affect the lipid barrier of the skin and may increase the risk of skin diseases, such as candidiasis, atopic dermatitis or acne [36]. All of the produced formulations in which the active substance was an aqueous apple extract with a complex of phenolic compounds had a pH value range of 6.03–6.98 at room temperature, which ensured their non-irritating effect on the skin.

The viscosity of semi-solid pharmaceutical forms affects the lubricity of the preparations, their contact with the skin, the release of the active compounds from the formulation and their penetration through the skin [41]. For these reasons, it was expedient to determine the viscosity of the modeled preparations. The results of the dynamic viscosity of the produced gels and emulgels containing aqueous apple extract are shown in Table 2. All of the produced formulations had a viscosity value range of 2.92–9.18 Pa·s at room temperature. The highest viscosity was observed for the G5 and G6 formulations, which contained 3% and 3.5% of carbomer, respectively. The lowest viscosity was observed for the G1 gel with a carbomer content of 0.5%. These results showed that the amount of extract in the formulation did not significantly affect the dynamic viscosity, but the viscosity in the gels and emulgels increased with increasing carbomer concentration in the test formulations. Ajazuddin et al. described that the prepared formulation showed variable viscosity, depending on both the concentration and type of the gelling material [22].

The avoidance of hypersensitivity or skin allergy from any of the ingredients is a major concern in the development of topical formulations and is dependent on their compatibility with the skin. When modeling gels and emulgels, carbomer 980 was selected as the gelling substance. Carbomer has been extensively used as the main drug-carrier for transdermal application. It has the advantages of high viscosity, high compatibility with other drugs, good thermal stability and excellent tissue compatibility [42]. Carbomer readily absorbs water, becomes hydrated and swells. Beside its hydrophilic nature, its cross-linked structure and its insolubility in water makes carbomer a potential candidate for use in delivery systems involving the controlled release of active substances. The quality and quantity of carbomer and other base concentrations were found to have a profound effect on the consistency of compositions [22]. The results of a study by Hayati et. al. showed that carbomer 940 is a nontoxic agent that improved tissue perfusion and decreased the area of necrotic tissue in burn wounds [42]. Therefore, the use of carbomer as the main ingredient for our prepared topical formulation is safe and devoid of any hazardous effects on skin. The selection of emulsifying materials is an important stage in the production of emulgels. A suitable emulsifier should have a reasonable balance between its hydrophilic and lipophilic groups and should be capable of producing stable emulsions [22]. Non-ionic surfactants, such as spans or tweens, have HLB (Hydrophilic Lipophilic Balance) values greater than 8 and are used in the formulation of oil-in-water emulsions, whereas mineral oils, such as liquid paraffin, have HLB values of less than 8 and are therefore employed in the formulation of water-in-oil emulsions. Emulgel was developed using Tween 20 as an emulsifier in its aqueous phase and Span 20 in its oil phase. Span 20 and Tween 20 mixtures contribute towards the greater stability of emulsions compared with pure Tween or Span systems [43].

In our study, all experimental gel and emulgel systems had a slightly yellow or dark yellow color, a homogeneous, soft, comfortable, non-oily texture without any visible particles and a base odor at room temperature. All of the produced formulations whose active substance was aqueous apple extract with a complex of phenolic compounds had pH values that are safe for use on the skin.

### 3.3. In Vitro Testing of the Release of Phenolic Compounds from Gels and Emulgels

The topical administration of formulations is a localized bioactive substance delivery system that can be used to introduce bioactive substances to any part of the body through ophthalmic, rectal, vaginal or skin routes [44]. The release of bioactive compounds from the pharmaceutical dosage form is important for absorption and therapeutic efficacy [45]. The release test is therefore a key measure for the evaluation of dosage form performance, both during the development of the formulation and for quality control purposes [46].

**Figure 2:**
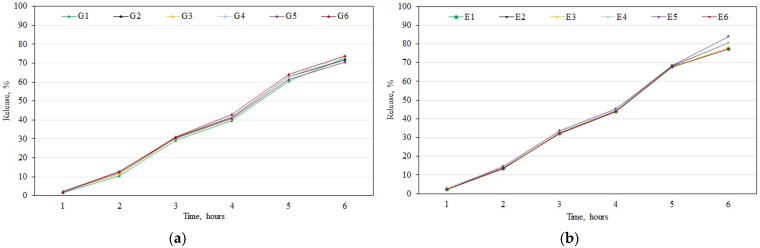
Percentage release of total phenolic compounds: (**a**) Percentage release of total phenolic compounds from gels G1–G6 after 6 h; (**b**) percentage release of total phenolic compounds from emulgels E1–E6 after 6 h (mean ± SD, *n* = 3).

After the analysis of the qualitative and quantitative composition of the biologically active compounds in the apple extracts, an in vitro release test of phenolic compounds from the gels and emulgels was performed during the next phase of the study. The test showed that the largest amounts of phenolic compounds were released from gels G1–G6 and emulgels E1–E6 after 6 h (Figure 2). We found that fewer phenolic compounds were released from the G1–G6 formulations (70.6–73.8%) compared to the amount (77.2–83.9%) released from the E1–E6 formulations (Figure 2). The largest amount (83.9%) of phenolic compounds was released from the E5 formulation (Figure 2, Panel b), while the smallest amounts (70.6%) were released from formulations G3 and G5 (Figure 2, Panel a). The in vitro release test showed that during the 1–4 h interval, both G1–G6 and E1–E6 formulations released statistically insignificant amounts of phenolic compounds, while after 5–6 h, larger amounts of phenolic compounds were released from the E1 –E6 formulations than from the G1–G6 formulations (Figure 2).

**Figure 3:**
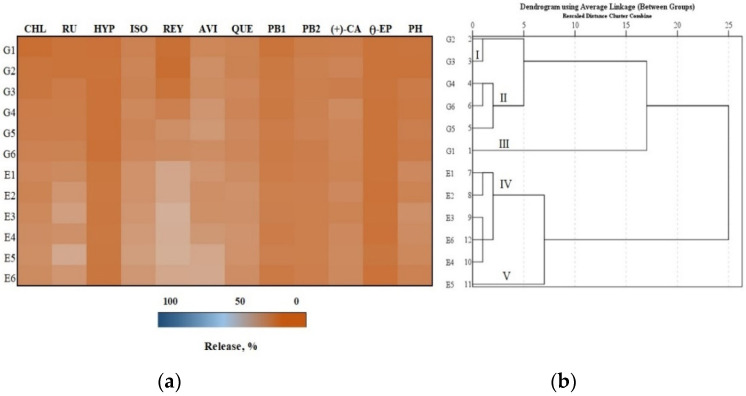
In vitro release test results: (**a**) Heatmap shows the percentage release of individual phenolic compounds from experimental gels (G1–G6) and emulgels (E1–E6) after 2 h; (**b**) the dendrogram illustrates variation in the total amount of phenolic compounds from experimental formulations after 2 h.

From the total amount of phenolic compounds released after 2, 4 and 6 h, the percentage release of the predominant biologically active compounds from the formulations was determined. Depending on the total amount released after 2, 4, and 6 h, a hierarchical cluster analysis was performed. We found that from the total amount of phenolics released after 2 h, avicularin was the predominant flavonoid (Figure 3, Panel a). The comparison of the amounts of individual phenolic compounds released from the test formulations showed that the largest amount (29.5%) of reynoutrin was released from the E5 formulation. Meanwhile, the lowest amount (7.7%) of chlorogenic acid was released from the G1 formulation compared to the amounts of the other individual phenolic compounds released from the test formulations (Figure 3, Panel a).

The total amount of phenolic compounds released from the gel and emulgel formulations after 2 h was divided into five clusters (Figure 3, Panel b). Formulations G2 and G3 were assigned to cluster I, which included larger amounts than the smallest total amounts of phenolics released. Formulations G4, G5 and G6 were assigned to cluster II, in which the average total amounts of the released phenolics were determined (12.4–12.9%). Formulation G1 was assigned to cluster III, in which the smallest total amount of phenolics was released (10.6%). Formulations E1, E2, E3, E4 and E6 were assigned to cluster IV, where smaller than maximum total amounts of phenolics were released. Formulation E5 was assigned to cluster V, which had the largest total amount of phenolics released (14.8%) (Figure 3, Panel b).

**Figure 4:**
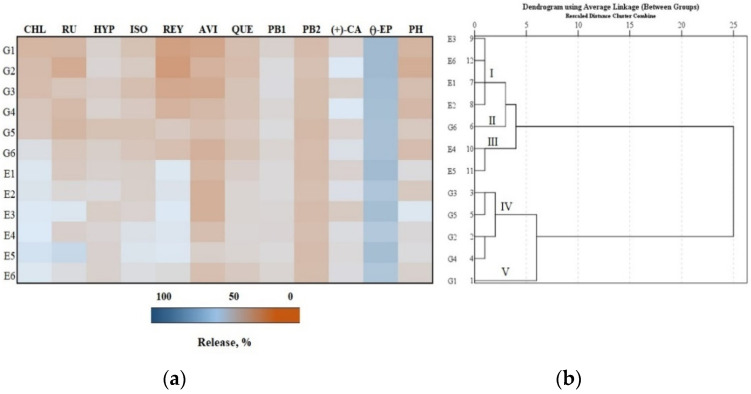
In vitro release test results: (**a**) Heatmap shows the percentage release of individual phenolic compounds from experimental gels (G1–G6) and emulgels (E1–E6) after 4 h; (**b**) the dendrogram illustrates variation in the total amount of phenolic compounds released from the experimental formulations after 4 h.

The flavan-3-ol group compound (–)-epicatechin was the predominant phenolic compound in the total amount of phenolic compounds released after 4 h (Figure 4, Panel a). We found that the largest amount (65.8%) of (–)-epicatechin was released from the G2 formulation compared to the amounts of the other individual phenolics released from the test formulations. The G2 formulation also released the smallest amount (22.8%) of reynoutrin compared to the amounts of other individual phenolic compounds released from the test formulations (Figure 4, Panel a).

The total amount of phenolic compounds released from the gel and emulgel formulations after 4 h was divided into five clusters (Figure 4, Panel b). Formulations E1, E2, E3 and E6 were assigned to cluster I, which had smaller than maximum amounts of total phenolics released. Formulation G6 was assigned to cluster II, where the average total amount of the released phenolics (43.0%) was found. Formulations E4 and E5 were assigned to cluster III, which had the largest total amounts of phenolics released (44.6% and 45.3%, respectively). Formulations G2, G3, G4 and G5 were assigned to cluster IV, which had smaller-than-average total amounts of phenolics released. Formulation G1 was assigned to cluster V, which had the smallest total amount of phenolics released (39.7%) (Figure 4, Panel b).

**Figure 5:**
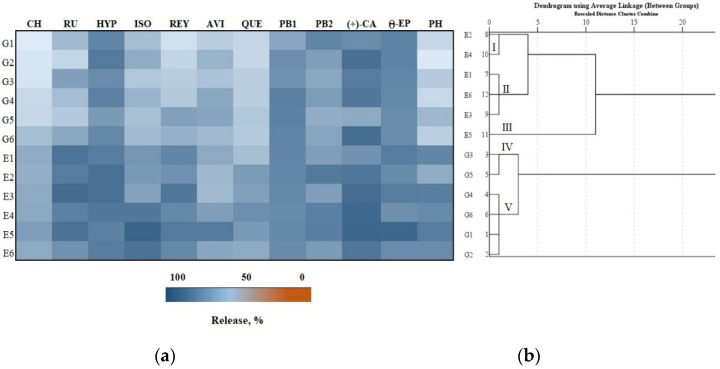
In vitro release test results: (**a**) Heatmap shows the percentage release of individual phenolic compounds from experimental gels (G1–G6) and emulgels (E1–E6) after 6 h; (**b**) the dendrogram illustrates variation in the total amount of phenolic compounds released from the experimental formulations after 6 h.

The maximum amounts of individual phenolic compounds were released from the gel and emulgel formulations after 6 h. Hyperoside and (+)-catechin were predominant phenolic compounds in the total amount of phenolic compounds released after 6 h (Figure 5, Panel a). We found that the largest amount (92.2%) of (+)-catechin was released from formulation E5 compared to the amounts of other individual phenolic compounds released from the test formulations (Figure 5, Panel a). Meanwhile, the smallest amount (50.3%) of chlorogenic acid was released from the G1 formulation compared to the amounts of other individual phenolic compounds released from the test formulations (Figure 5, Panel a).

The total amount of phenolic compounds released from the gel and emulgel formulations after 6 h was divided into five clusters (Figure 5, Panel b). Formulations E2 and E4 were assigned to cluster I, where the total amounts of phenolic compounds released were found to be smaller than the maximum. Formulations E1, E3 and E6 were assigned to cluster II, in which the average total amount of phenolic compounds released (77.2–78.2%) was found. Formulation E5 was assigned to cluster III, which had the largest total amount of phenolic compounds released (83.9%). Formulations G3 and G5 were assigned to cluster IV, which had the smallest total amounts of phenolic compounds released (70.6%). Formulations G1, G2, G4, and G6 were assigned to cluster V, which had smaller than average total amounts of phenolic compounds released (Figure 5, Panel b).

The in vitro release test showed that after 2 h, the average amounts of phenolic compounds released from the test formulations were from 10.6% to 14.8%; after 4 h, these amounts ranged from 39.7% to 45.3% and after 6 h they ranged from 70.6% to 83.9%. Larger amounts of individual phenolic compounds were released from the emulgel formulations than from the gel formulations. The physicochemical properties of the investigated gels and emulgels might have influenced the amount of phenolic compounds released as well as the release kinetics. Emulgel helps in the incorporation of hydrophobic bioactive substances into the oil phase; oil globules are then dispersed in the aqueous phase resulting in an oil-in-water emulsion and, consequently, this emulsion can be mixed into the gel base. This may provide better stability and release of bioactive substances than by simply incorporating drugs into the gel base [47]. Pradhan et al. mentioned that bioactive substance release from emulgels is controlled by the interactions between drug and surfactant and the partitioning of the active substance between the oil and the water phases [48]. Elbayoumi and Torchilin stated that emulgel contains the properties of both emulsions and gels and that it acts as a dual controlled release system [49]. The impact of the gelling material on the release rate of the active substance from emulgels has also been studied. Ajazuddin et al. estimated that there is an inverse correlation between the content of the gelling material and the amount of the active substance released [22].

### 3.4. Antioxidant Activity In Vitro

Evaluating phenolic compounds’ antiradical and reducing potential is relevant to determining the total antioxidant effects by using different spectrophotometric assays. This makes it possible to obtain detailed data on the antioxidant effects of their multi-component matrices. To achieve this, the antioxidant effect was evaluated in the gel (G1–G6) and emulgel (E1–E6) formulations after 2, 4 and 6 h and in 1% apple extract (AE) by applying two in vitro assays, namely, DPPH and FRAP. The evaluated antioxidant mechanisms have relevent implications that may be used for a better understanding of the protective activities of bioactive compounds in apple extracts against acute or chronic diseases.

**Figure 6:**
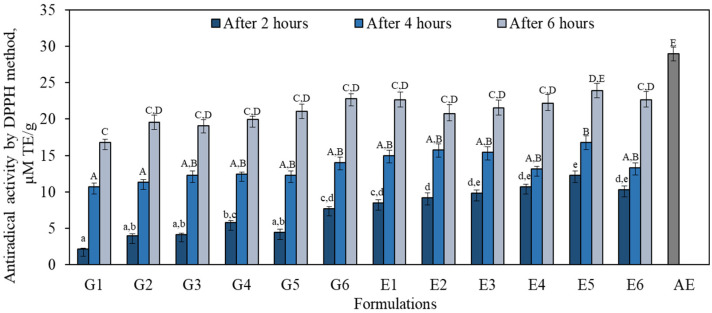
Antiradical activity evaluated in gel (G1–G6) and emulgel (E1–E6) formulations and in 1% apple extract (AE) by the DPPH method. The means followed by different uppercase and lowercase letters are significantly different at *p* < 0.05.

We found that the antiradical activity in the gel (G1–G6) and emulgel (E1–E6) formulations after 2, 4 and 6 h and in 1% apple extract ranged from 2.1 μM TE/g to 29.0 μM TE/g (Figure 6). The strongest statistically significantly antiradical activity evaluated by the DPPH method (29.0 μM TE/g) was found in the 1% apple extract. Faramarzi et al. determined that the antiradical activities of scavenging DPPH free radicals ranged from 10.1 to 129.1 µM TE/g among 67 apple extracts [50]. The weakest DPPH free radical scavenging activity in all the gel (G1–G6) and emulgel (E1–E6) formulations was observed after 2 h, compared to the activity observed in the formulations after 4 or 6 h. After 2 h, the strongest statistically significantly antiradical activity (12.3 ± 0.6 μM TE/g) was found in the E5 formulation, compared to the activity found in formulations G1–G6, E1 and E2. After 4 h, the weakest antiradical activity was observed in formulations G1 and G2 (10.7 ± 0.5 μM TE/g and 11.3 ± 0.5 μM TE/g, respectively), which was statistically significantly different from the activity determined in formulation E5 and in 1% AE. After 6 h, all the gel (G1–G6) and emulgel (E1–E6) formulations demonstrated stronger DPPH free radical scavenging activity compared to the activity found in the formulations after 2 or 4 h. After 6 h, the strongest antiradical activity (23.9 ± 1.2 μM TE/g) was found in formulation E5 (Figure 6).

**Figure 7:**
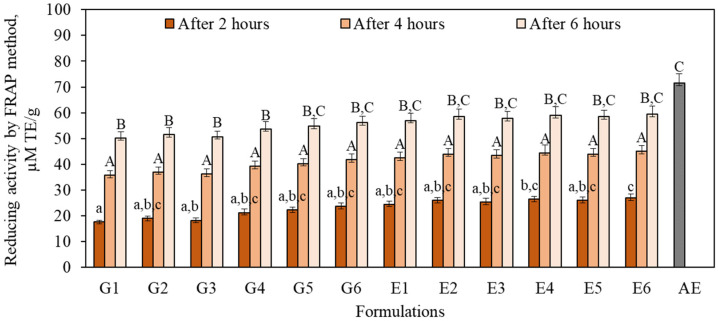
Reducing activity evaluated in gel (G1–G6) and emulgel (E1–E6) formulations and in 1% AE by the FRAP method. The means followed by different uppercase and lowercase letters are significantly different at *p* < 0.05.

The reducing activity determined by the FRAP method in the gel (G1–G6) and emulgel (E1–E6) formulations after 2, 4 and 6 h and in the 1% apple extract ranged from 17.8 μM TE/g to 71.5 μM TE/g (Figure 7). The strongest reducing activity evaluated by the FRAP method (71.5 μM TE/g) was found in 1% AE. Xu et al. found that the reducing activity found by the FRAP method in apple flesh and peel samples varied from 36.5 μM TE/g to 75.9 μM TE/g [51]. Previous data showed that in 13 apple cultivars, FRAP values varied between 50.5–192.0 and 71.8–137.7 µM TE/g in the flesh and the peel, respectively [52]. After 2 h, all the gel (G1–G6) and emulgel (E1–E6) formulations had weaker reduction activity, evaluated with the FRAP method, compared with the activity found in the formulations after 4 or 6 h (Figure 7). After 4 h, the reducing activity determined by the FRAP method in all the gel (G1–G6) and emulgel (E1–E6) formulations did not differ with any statistical significance. Stronger reducing activity was observed in all the gel (G1–G6) and emulgel (E1–E6) formulations after 6 h compared to the activity found in the formulations after 2 or 4 h. After 6 h, the strongest reducing activity (59.9 ± 2.9 μM TE/g) was found in formulation E6 (Figure 7).

The higher amounts of phenolic compounds released from the tested gel and emulgel formulations possibly contributed to the stronger antiradical and reducing activity after 6 h compared to the activity observed in the formulations after 2 or 4 h. Scientific research provides data suggesting that antioxidant effects are positively correlated with the levels of phenolics [53]. Li et al. stated that high concentrations of phenolic compounds exhibit high antioxidant potential [54]. The analysis of the results of the in vitro release test after 6 h showed that the highest amount released from the test formulations compared to the amounts of other individual phenolic compounds was that of (+)-catechin. According to previous research, (+)-catechin and quercetin are the main phenolic compounds that promote to the antioxidant activities of apple samples [54].

Currently, there is great consumer interest in natural products, including compounds derived from fruits, plants and herbs, which are rich in antioxidants, such as phenolic compounds. Phenolic compounds are an important group of secondary metabolites in regulating the damage that is potentially sustained by biological molecules, such as DNA, protein, lipids and body tissue, in the presence of reactive species. Abnormally low levels of antioxidants have been associated with impaired wound healing [5]. Phenolic acids, such as chlorogenic acid, demonstrate their strong antioxidant properties by increasing the activity of superoxide dismutase, catalase and glutathione and decreasing lipid peroxidation. Topical forms with chlorogenic acid can induce the process of wound healing due to the acid’s antioxidant activities, its ability to increase collagen synthesis and its regulation of mediator functions in different phases of the wound healing process [55]. It has been shown that antioxidants can increase wound healing, especially in chronic wounds [51]. In our study, we evaluated the radical scavenging activity and the metal ion chelation ability of gel and emulgel formulations containing a phenolic compound complex from apple extracts. Phenolic compounds, which are abundant in apple extract, may be an interesting choice of active substance for topical formulations used for treating skin damage (for example, scars or wounds).

Sun protection, as in sunscreens that absorb harmful UV radiation, is the first and foremost strategy to avoid photodamage to the skin [56]. Previous studies revealed that plant extracts rich in phenolic compounds have the ability to absorb UV radiation and may be potential natural sunscreens in cosmetic formulations, helping to avoid photo-induced skin damage [57]. Phenolic compounds have photoprotective properties that are associated with antioxidant activities, such as their capacity to chelate iron, which may harm lipids and proteins in cell membranes. Similarly, phenolics can regulate some signaling pathways. One way in which they do this is through their inhibition of xanthine oxidase, which is considered as a source of reactive oxygen species (ROS), which are the most widespread free radicals and contribute to oxidative stress [58,59]. The topical formulations with apple extract rich in the phenolic compound complex used in our study are a component of natural origin that could be used in cosmetic products to protect against UV radiation, premature aging and sun-induced skin hyperpigmentation. The phenolic compounds with strong antioxidant properties that are abundant in apple extract may be an interesting choice of active substance for topical formulation and the development of pharmaceutical forms or cosmetics for the treatment of skin damage. Examples of its application would include the promotion wound healing or the protection of skin from UV radiation.

## 4. Conclusions

In our study, we found that in the apple extract from the ‘Paprastasis antaninis’ cultivar, the compounds of the flavan-3-ol group predominated among all the identified and quantified phenolic compounds. The in vitro release test revealed that the largest total amount of phenolics was released from formulation E5 and that the smallest amount was released from formulations G3 and G5. We also determined the percentage of the individual phenolic compounds from the total amount of phenolics released from the formulations after 2, 4 and 6 h. We found that avicularin was the predominant flavonoid among the total amount of phenolic compounds released after 2 h. The flavan-3-ol group compound (–)-epicatechin was the predominant phenolic compound among the total amount of phenolic compounds released after 4 h. The largest amounts of individual phenolic compounds were released from the gel and emulgel formulations after 6 h. Hyperoside and (+)-catechin were the predominant phenolic compounds among the total amount of phenolic compounds released after 6 h. The antiradical activity evaluated by the DPPH method and the reducing activity determined by the FRAP method observed in all the gel (G1–G6) and emulgel (E1–E6) formulations after 6 h were stronger compared to when these activities were observed in the formulations after 2 or 4 h. The higher amounts of phenolic compounds released from the tested gel and emulgel formulations possibly contributed to the stronger antioxidant activity after 6 h compared to the activity observed in the formulations after 2 or 4 h. Gels and emulgels rich in apple extracts have strong antioxidant properties and may be a promising choice for the development of new, innovative pharmaceutical forms or cosmetics.

## Figures and Tables

**Table 1:** Composition of the gels, emulsions and emulgels.

Composition No.	Carbomer, g	NaOH, mL	Glycerin, g	Castor Oil, g	Span 20, g	Tween 20, g	Water, g	Content, g
Gels								
G1	0.5	3–4 drops	-	-	-	-	ad 100	100 ± 0.5
G2	1.0	3–4 drops	-	-	-	-	ad 100	100 ± 0.5
G3	2.0	3–4 drops	-	-	-	-	ad 100	100 ± 0.5
G4	2.5	3–4 drops	-	-	-	-	ad 100	100 ± 0.5
G5	3.0	3–4 drops	-	-	-	-	ad 100	100 ± 0.5
G6	3.5	3–4 drops	-	-	-	-	ad 100	100 ± 0.5
Emulsion								
N8	-	-	10	10	6	6	ad 100	100 ± 0.5
Emulgels								
E1	Mixed G1 and N8 (1:1)							100 ± 0.5
E2	Mixed G2 and N8 (1:1)							100 ± 0.5
E3	Mixed G3 and N8 (1:1)							100 ± 0.5
E4	Mixed G4 and N8 (1:1)							100 ± 0.5
E5	Mixed G5 and N8 (1:1)							100 ± 0.5
E6	Mixed G6 and N8 (1:1)							100 ± 0.5

**Table 2:** Organoleptic characteristics and physicochemical parameters of gels and emulgels with 1% AE.

Formulations						
Gel						
Composition No.	G1	G2	G3	G4	G5	G6
Organoleptic characteristics	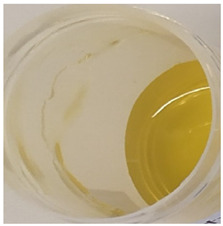	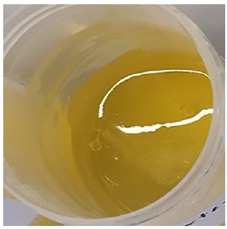	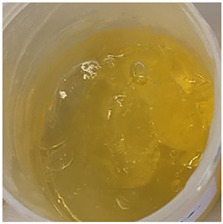	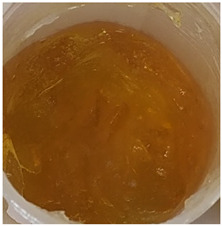	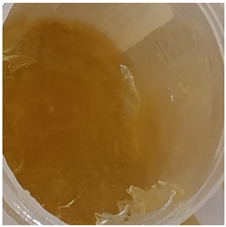	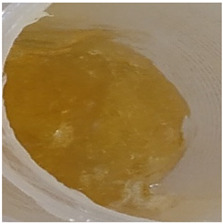
	Yellow homogeneous gel with base odor			Dark yellow homogeneous gel with base odor		
pH value (*n* = 3)	6.60 ± 0.34	6.67 ± 0.34	6.72 ± 0.35	6.71 ± 0.35	6.76 ± 0.35	6.76 ± 0.35
Viscosity (Pa·s, *n* = 3)	2.92 ± 0.15	7.74 ± 0.39	7.96 ± 0.40	8.25 ± 0.41	9.03 ± 0.55	9.18 ± 0.46
Emulgel						
Composition No.	E1	E2	E3	E4	E5	E6
Organoleptic characteristics	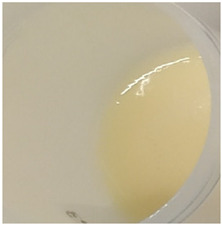	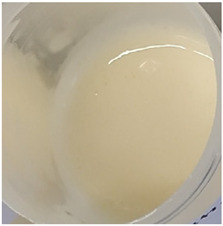	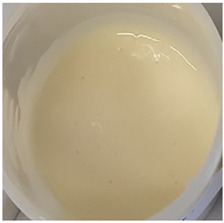	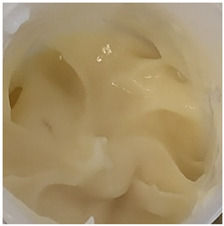	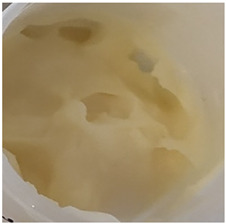	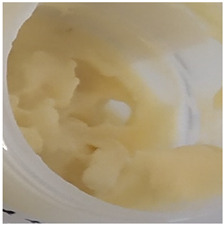
	Homogeneous emulgel with slightly yellow color, base odor, non-oily.					
pH value (*n* = 3)	6.04 ± 0.30	6.03 ± 0.30	6.14 ± 0.30	6.16 ± 0.30	6.20 ± 0.30	6.27 ± 0.30
Viscosity (Pa·s, *n* = 3)	2.94 ± 0.15	7.51 ± 0.38	7.68 ± 0.38	8.01 ± 0.40	8.97 ± 0.45	8.98 ± 0.45

## Data Availability

All the datasets generated for this study are included in the article.
